# The Best Means of Advancing Dental Science

**Published:** 1862-09

**Authors:** J. Allen


### THE BEST MEANS OF ADVANCING DENTAL SCIENCE.

BY DR. J. ALLEN.

An Address delivered on retiring from the Presidency of the American Dental Convention, held at Trenton Falls, August 8th, 1862.

Members of the American Dental Convention:—Another year has passed since our last annual meeting, which has been fraught with events, long to be remembered in the history of our country.

The efforts which have been made to sever the bonds of our Union, overthrow this government, divide and alienate us as a people, tell upon our profession, as seen by the absence of many Southern members, who were wont to be with us, and take part in our deliberations, in years gone by; but we trust the time is not far distant when we shall again be united, and all work together in promoting the best interests of our profession and of our common country.

At our last annual meeting, it was my privilege to make some remarks upon the causes which retard dental progress. I will now present a few thoughts upon the best means (as I conceive) of advancing dental science. This can be done by two distinct methods: The one by the united efforts of the profession, and the other by individual effort.

Let the dental practitioners throughout the country act in concert in building up our dental colleges; let them encourage more liberally our dental journals; establish and sustain properly organized dental associations: and by these means dental science will be advanced. I need not detail the advantages that each of these possess in diffusing dental knowledge among our profession—they are self-evident to every thinking mind. I will, therefore, dwell more particularly upon individual efforts, as a means of advancing dental science; for we should not only avail ourselves of all that can be taught and learned from others, but we often find it necessary to draw largely upon our own mental resources, to enable us to accomplish our purposes; for such is the peculiar nature of our profession that every successful practitioner must rely upon himself to devise ways and means to meet the various exigencies that are constantly presenting themselves in dental practice. Cases frequently occur, for which he may seek in vain to find in books exact parallels to those requiring his aid, He must then fall back upon his individual resources to guide him in the accomplishment of the object to be attained. It is, therefore, necessary that he should know himself; he should know his own mental organism and its powers, as well as those of his corporeal system.

The human senses, as every one knows, are five in number, and each communicates its proper sensation. It is by the eyes that we see, by the ears that we hear, by the nose that we smell, by the tongue and palate that we taste, and by the sense of touch that we feel; and every anatomist knows that in order to produce sensation upon any one of these senses, the impression is first made upon the organ; the organ serves only as a medium to convey it to the nerves, and the nerves serve as a medium to carry it on to the brain. Here the corporeal part ends; the rest is all intellectual. Here we enter another field of observation, and we discover that the mind has the power of turning its attention upon itself, and there learns the nature, manner and power of its own operations.

There are certain faculties of the mind entirely different from the five senses, such as perception, thinking, reasoning, knowing, willing, and also all the different energies and passions of the mind. It is to these intellectual faculties that we invite special attention; for herein lies the great power of advancing all sciences. It is the power derived from the exercise of these faculties that man has been able to devise means by which he can fathom the depths of the ocean, determine the diameter and circumference of this and other worlds, determine the distances of the various planets from each other, and the momentum with which they travel thro' space.

It was through his individual efforts that Herschel was enabled to extend his astronomical observations far beyond those of his predecessors, and bring to view other planets, previously unknown to man. It was through the medium of his own mind that Rittenhouse first calculated eclipses on his plow-handle. It was the individual efforts of Morse (aided, perhaps, by other suggestions, comparativaly insignificant), that enabled him to develop the principle by which ideas can be transmitted with lightning speed from one distant point to another. It was by the strength of his own reasoning powers that Columbus discovered the American continent.

These, and many other examples that might be given, show the wonderful power and capacities of the human mind to work out things which would appear to casual observers as impossibilities; but the human mind is so constituted that it can work upon and within itself, and by this means important principles can be brought to light. This is the central thought that we wish to present with reference to individual efforts as a means of advancing dental science. Man, know thyself, is an injunction applicable to each one of us; our Creator has endowed us with mental faculties, which we ought to understand, and we should study them assiduously; we should cultivate and strengthen them, that they may be relied upon when brought into requisition. To advance beyond our present stand-point, we should first acquire a thorough knowledge of that which is now known and rendered available through the medium of books, colleges, associations, etc. And, when these external resources shall have become exhausted, then direct the thoughts inwardly, amid the hidden recesses of the mind, with a view to discover still higher practical points than those acquired from other sources.

Gibbon says, "Every person has two educations—one which he receives from others, and one more important, which he gives to himself." It is this self-culture that should be relied upon more than any other for advancement, for if none go beyond where others have gone, there will be no advance. It is the neck ahead that wins the race.

This brings us to the consideration of original ideas, or those which may have been suggested to the mind by some external object or circumstance. In either case, they should be fostered with care. Good ideas often perish for want of nourishment. In such cases, there has not been a sufficient amount of reflection bestowed upon them to mature the conception. In order to develop an idea, it should be cultivated with patience; it should be cared for, turned and re-turned in every direction, looked at in all its aspects, placed in its various relations, scrutinized in all its dimensions and proportions, then clothed with a body. After this is done, then test its merits; note the results; strengthen the weak points; devise means to overcome unforseen difficulties. "Labor and wait" until sufficient time shall have elapsed to fully develop the principle involved.

When an idea is first conceived, it is usually vague and chaotic—a mere germ; but by revolving it over and over again in the mind, it gradually unfolds like a thing of life, and we then begin to see whether it possesses merit and practicability. If we can discover that it does, we should not let it flit away again void as it came to us, but carefully nurture and cherish it, for it may develop a principle of great importance. You recollect when Franklin discovered the identity of lightning and electricity, it was sneered at, and people asked, "Of what use is it?" To which he replied, "What is the use of a child? it may grow to be a man!" Give heed to these little silent messengers called ideas, that gently come gliding into the mind, for they may prove to be of incalculable value. They may reveal important principles not to be found elsewhere. It was these silent whisperings that disclosed to Newton the principles of astronomy! To Archimedes the means of determining the distances between the different planets! and to Fulton how to propel the steamboat. It was an idea that enabled Whitney to make the cotton gin, and Jenner to prevent smallpox by vaccination. Thousands of instances might be given in every branch of science and art, where individuals, by their own efforts, have made valuable discoveries or improvements, by simply carrying out practical ideas. True, in many instances they were attended with great labor and expense, for it is only the bare idea that first comes into the mind; the minute details all have to be wrought out by those who receive them; but the ends, when accomplished, have fully justified the means employed. The man who becomes the humble instrument for developing an important principle will often meet with difficulties and trials almost insurmountable; but by keeping his mind's eye steadily fixed upon the idea as his pole-star, and gradually working his way onward by the light which it reflects, he may have the pleasing satisfaction of seeing his efforts ultimately crowned with success.

Newton, on being asked by what means he had worked out his extraordinary discoveries, replied, "By always thinking unto them." At another time he said, "I keep the subject continually before me, and wait till the first dawnings open slowly, by little and little, into a full and clear light." In these few words he has given us the key with which to unlock the citadel of the mind, and bring out hidden treasures that might otherwise have remained concealed. There are those who conceive good ideas, but their restless spirits will not permit them to wait patiently, and labor on perseveringly, until practical results are obtained, and they abandon them, because of the slow progress of their development. Such men have not the patience and perseverance which is necessary to change the mulberry leaf into satin. "Alas!" said a good old lady, speaking of her brilliant but careless son, "he has not the gift of continuance." This may be said of many who have strong powers of conception, but their working qualities are not well trained, and their ideas perish in darkness.

> "See first that the design is wise and just,
> That ascertained, pursue it resolutely;
> Do not for one repulse forego the purpose,
> That you resolved to effect."

Let each member of our profession apply these principles to himself, and put forth his own personal efforts with a view of doing something that will tend to advance our cause.

"It is not enough," said John Locke, "to cram ourselves with a great load of collections; unless we chew them over again, they will not give us strength and nourishment. That which is put into us by others is always far less ours than that which we acquire by our own diligent and persevering efforts. Knowledge, conquered by labor, becomes a property entirely our own, and of great practical importance to those who possess it."

"Knowledge dwells
In heads replete with thoughts of other men;
Wisdom in minds attentive to their own
Knowledge, a rude, unprofitable mass,
The mere materials with which wisdom builds,
Till smoothed and squared and fitted to its place,
Does but incumber whom it seems t' enrich."

In conclusion, allow me to return to you my sincere thanks for the kind forbearance and attention you have extended towards your retiring President, during his official term of service with you. And may that spirit of harmony and unanimity which has heretofore characterized your deliberations continue to be a prominent feature in all of your subsequent meetings. And may your future discussions not be of that kind which leaves the heart cold and the head empty; but may they teem with wisdom's words, and fill the mind with mental food. And when long years shall have passed away, may we still look back with fond recollections upon these annual convocations, as some of the green and pleasant spots in the horizon of our memories.

